# Supplementary material for: Design, Synthesis and Pharmacological Evaluation of New Quinoline-Based Panx-1 Channel Blockers
Source: Int J Mol Sci. 2023 Jan 19;24(3):2022. doi: 10.3390/ijms24032022 (PMC9916356; doi:10.3390/ijms24032022)
Supplement: Supplementary file 1 [file ijms-24-02022-s001.zip › ijms-2116241-supplementary.pdf]

Supplementary Materials

for

Design, synthesis and pharmacological evaluation of new quinoline-based Panx-1 channel blockers

Letizia Crocetti [1], Maria Paola Giovannoni [1], Gabriella Guerrini [1], Silvia Lamanna [1], Fabrizio Melani [1], Gianluca Bartolucci [1], Marco Pallecchi [1], Paola Paoli [2], Martina Lippi [2], Junjie Wang [3] and Gerhard Dahl [3]*

[1] Neurofarba, Pharmaceutical and Nutraceutical Section, University of Florence, Via Ugo Schiff 6, 50019, Sesto Fiorentino, Italy

[2] Department of Industrial Engineering, University of Florence, Florence, Italy

[3] Department of Physiology and Biophysics University of Miami School of Medicine 1600 N.W. 10th Avenue Miami, Florida 33136

***Corresponding Author**

Dr. Gabriella Guerrini

Department of Neurofarba,

University of Florence, Via Ugo Schiff 6,

Sesto Fiorentino, Firenze 50019, Italy.

Tel. +39 055 4573766

Email: gabriella.guerrini@unifi.it

Table of Contents:

1. Synthesis of reaction intermediates
2. NMR spectra (^{1}H, HSQC,HMBC) of some reaction intermediates (**4c**, **4e**, **5c**, **5e**) and NMR spectra (^{1}H and ^{13}C) of some representative final compounds (**6a**, **6d**, **6g**, **10**, **13c**, **13d**, **13f**, **13g** and **15**)
3. Chemical stability tests (**Tables S1–S4** and **Figures S1–S6**).
4. Single-Crystal X-ray Diffraction (**Table S5** and **Figure S7**).

5. ADMET assessment (**Tables S6–S11**).
6. Elemental analysis (**Table S12**).

1. Synthesis of reaction intermediates

The scheme describes the procedures used for the synthesis of some reagents (others are commercially available) to obtain the final compounds. For reagents **3a-c**, the synthetic process involves alkylation of commercial 4-nitrobenzenesulfonamide (**1**) with the appropriate halide derivative and K_2CO_3 in dry DMF. Specifically, 1-bromopropane is used to obtain the monoalkylated and bialkylated products **3a,b** (Carmellino, Caccialanza, & Borgogna, 1983) and benzyl bromide is used to obtain the bialkylated product **3c** (Hui Po & Sheu Ju, 1987). Finally, for compound **7**, the synthetic process involved three steps: esterification of commercial 4-sulfamoylbenzoic acid (**4**) resulting in the corresponding ethyl ester 5 (Turkes, et al., 2019); alkylation of the sulfonamide with benzyl bromide (using the same procedure reported above for reagents 3a-c) leading to the dibenzylated intermediate **6** (Caturla, et al., 2004); and finally, hydrolysis of the ester to acid **7** (Hanke, et al., 2013).

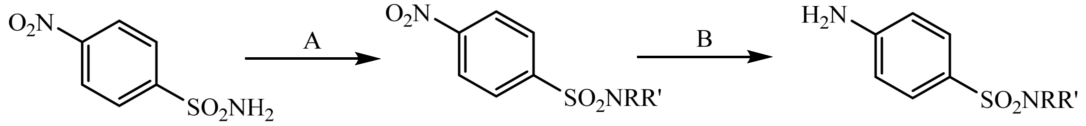

1 **2a-c** **3a-c**

2,3	R	R'
**a**	H	$CH_2CH_2CH_3$
**b**	$CH_2CH_2CH_3$	$CH_2CH_2CH_3$
**c**	CH_2Ph	CH_2Ph

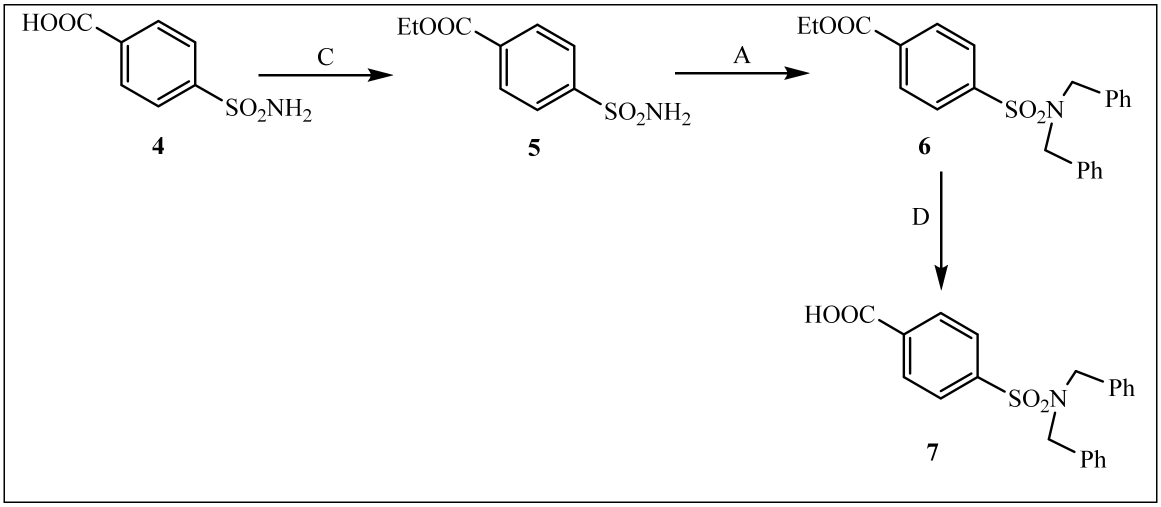

Reagent and conditions:

A: K_2CO_3, dry DMF, r.t., 30′, then appropriate halide derivative, 80°C, 3h.

B: H_2, Pd/C, EtOH abs, 30 psi, r.t., 1h 30′.

C: EtOH abs, H_2SO_4 conc., reflux, 5h.

D: EtOH 96%, NaOH 40%, reflux, 2h.

2. NMR spectra of some reaction intermediates and representative final compounds

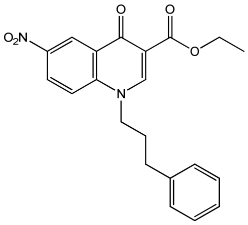

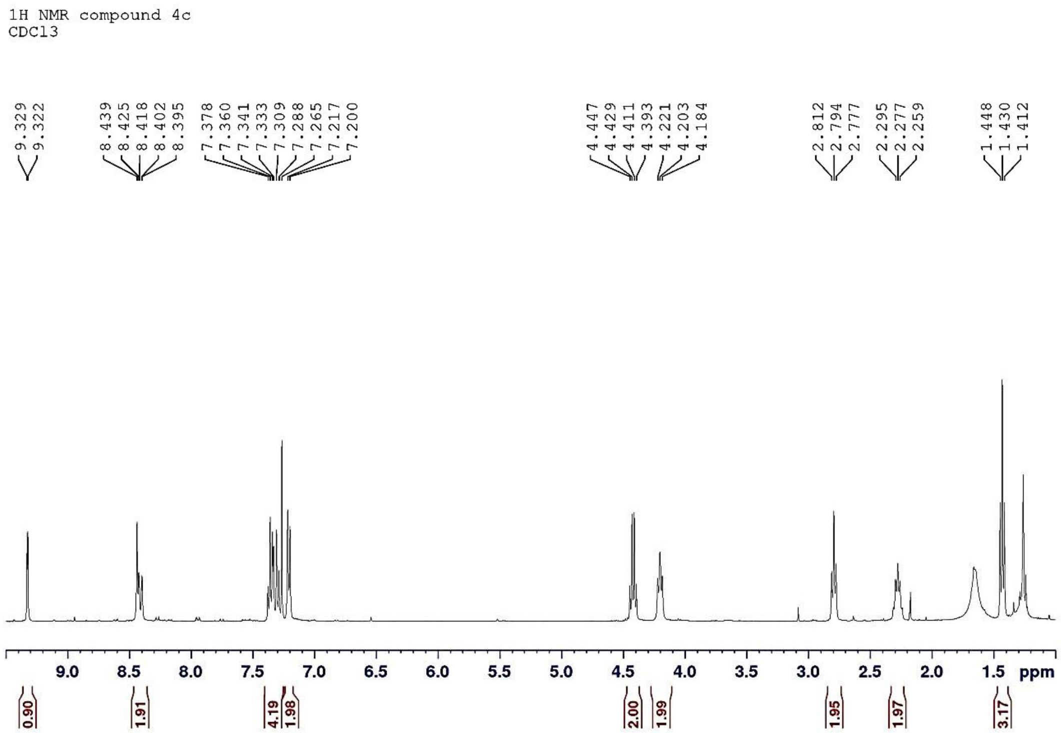
1H NMR compound 4c
CDCl3
9.329
9.322
8.439
8.425
8.418
8.402
8.395
7.378
7.360
7.341
7.333
7.309
7.288
7.265
7.217
7.200
4.447
4.429
4.411
4.393
4.221
4.203
4.184
2.812
2.794
2.777
2.295
2.277
2.259
1.448
1.430
1.412
9.0 8.5 8.0 7.5 7.0 6.5 6.0 5.5 5.0 4.5 4.0 3.5 3.0 2.5 2.0 1.5 ppm
0.90
1.91
4.19
1.98
2.00
1.99
1.95
1.97
3.17

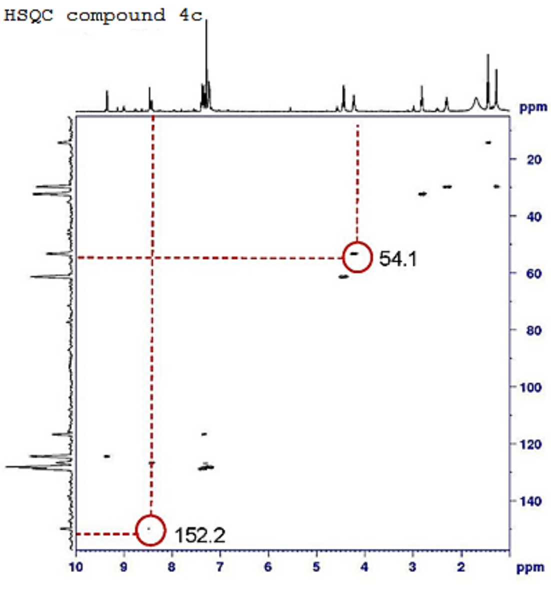
HSQC compound 4c
54.1
152.2
ppm

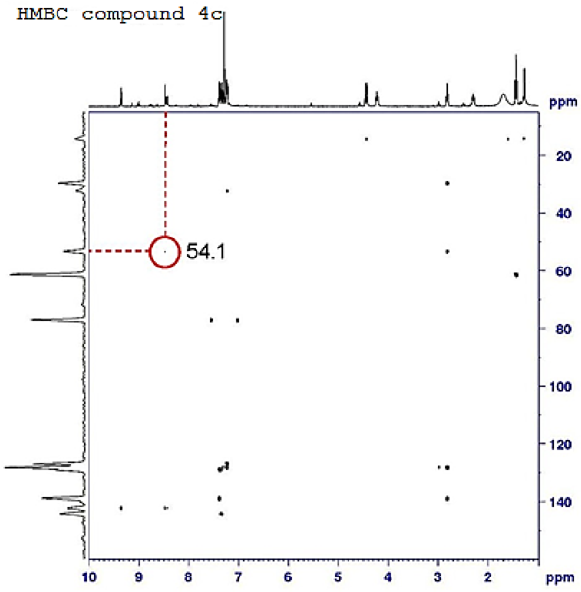
HMBC compound 4c
54.1
ppm

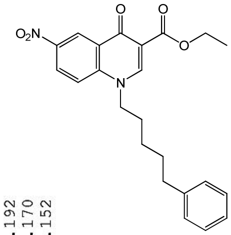

1H NMR compound 4e
CDCl3

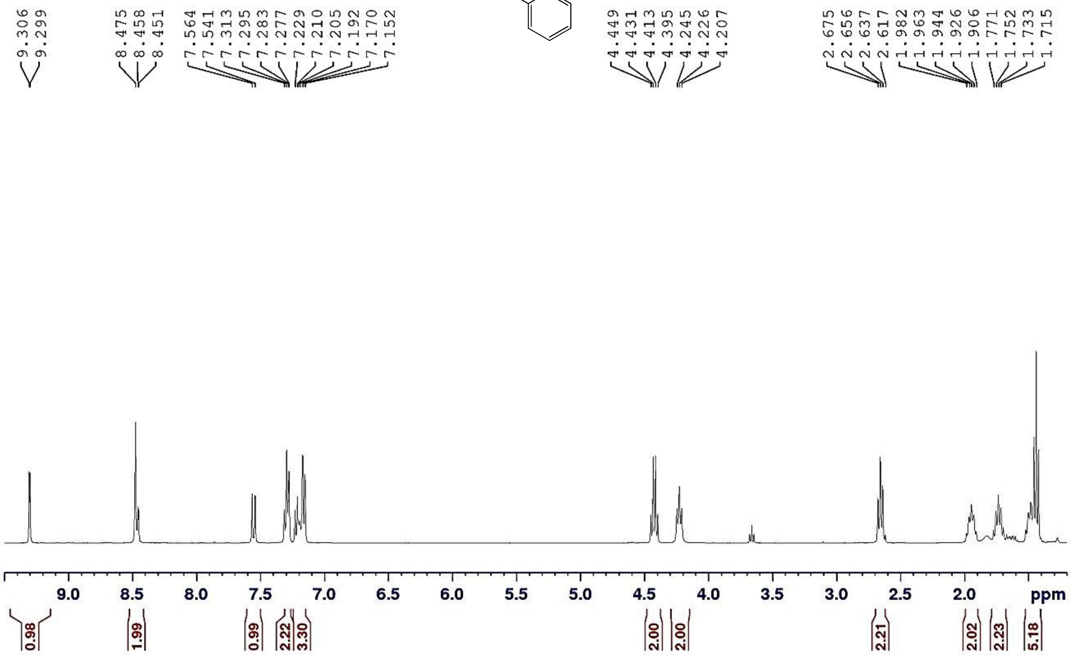

HSQC compound 4e

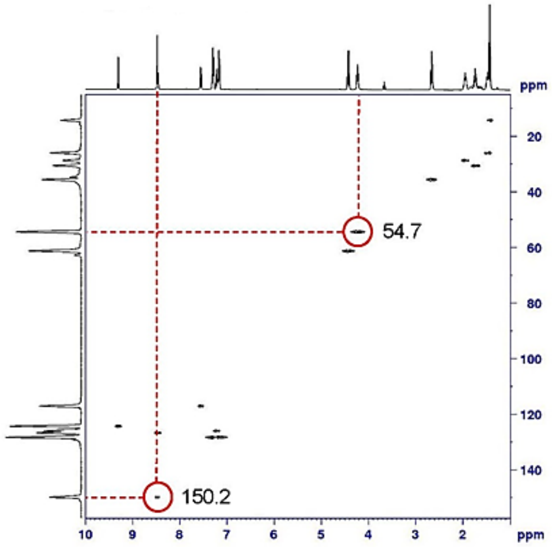

HMBC compound 4e

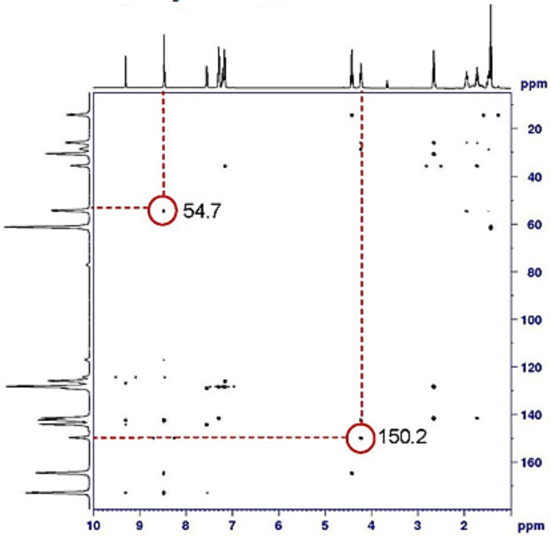

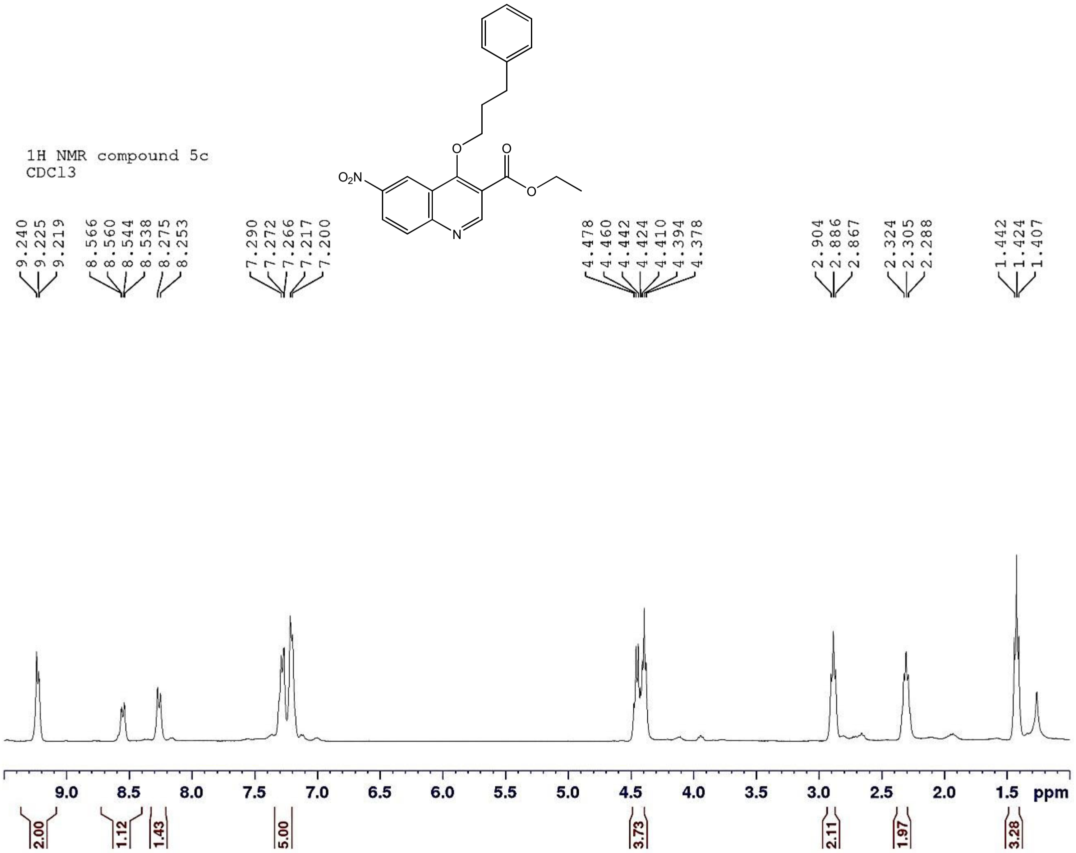
1H NMR compound 5c
CDCl3
O2N
O
O
O
N
9.240 9.225 9.219 8.566 8.560 8.544 8.538 8.275 8.253 7.290 7.272 7.266 7.217 7.200 4.478 4.460 4.442 4.424 4.410 4.394 4.378 2.904 2.886 2.867 2.324 2.305 2.288 1.442 1.424 1.407
9.0 8.5 8.0 7.5 7.0 6.5 6.0 5.5 5.0 4.5 4.0 3.5 3.0 2.5 2.0 1.5 ppm
2.00 1.12 1.43 5.00 3.73 2.11 1.97 3.28

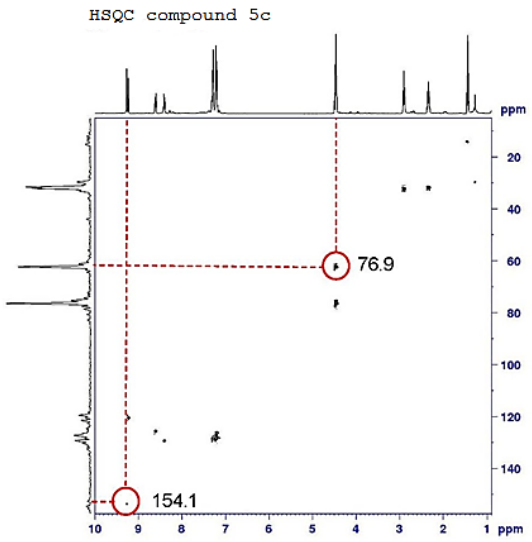
HSQC compound 5c
76.9
154.1
ppm

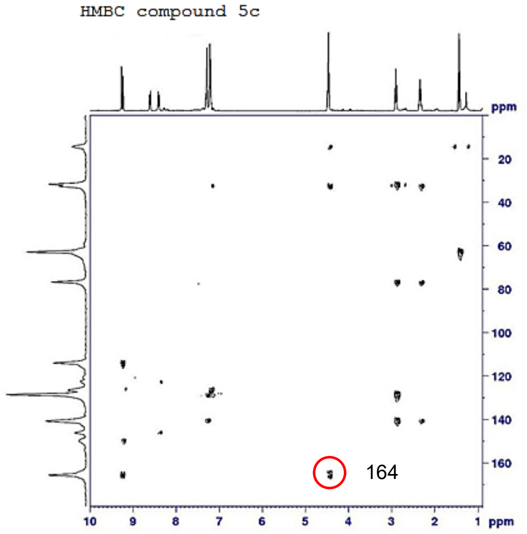
HMBC compound 5c
164
ppm

1H NMR compound 5e
CDCl3

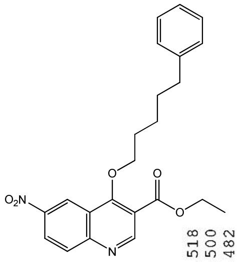

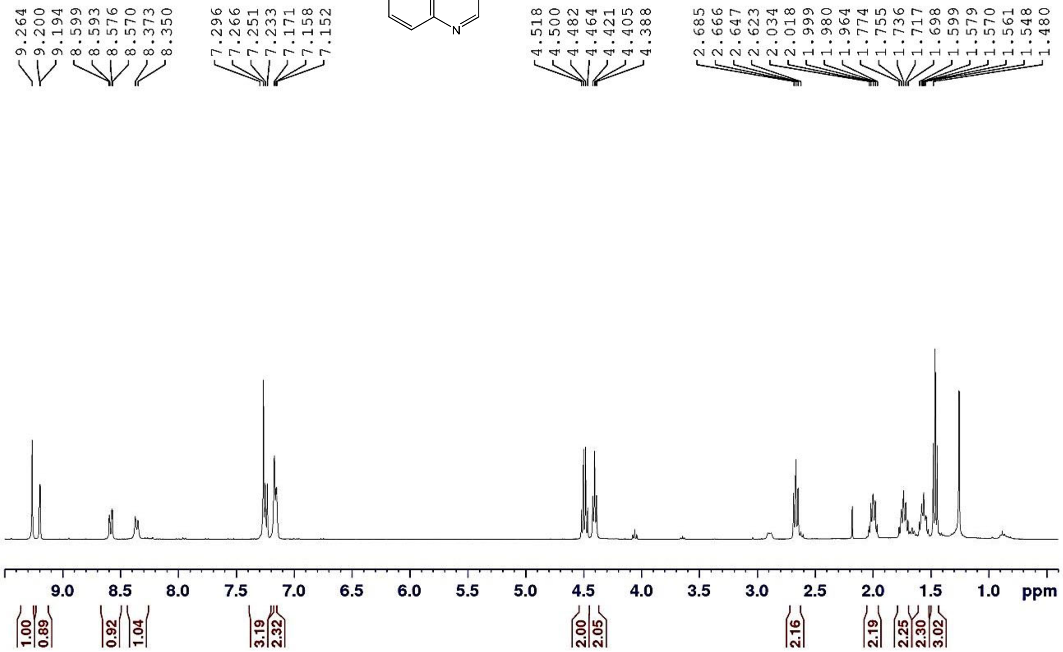

HSQC compound 5e

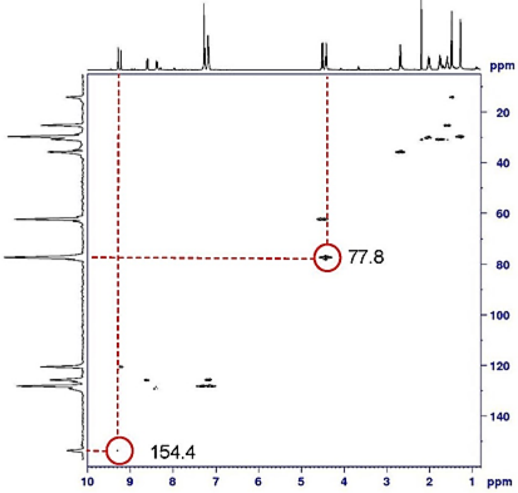

HMBC compound 5e

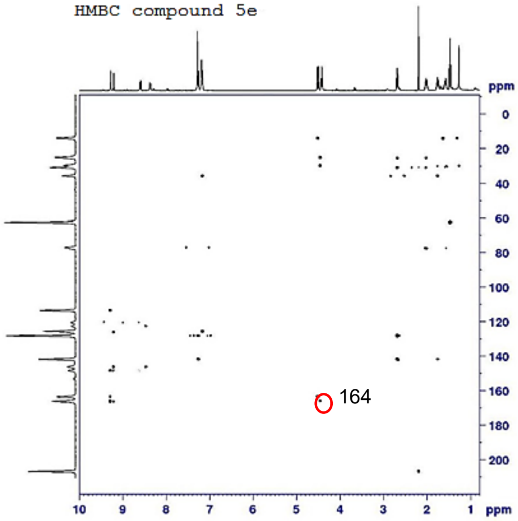

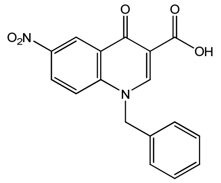

1H NMR compound 6a
DMSO

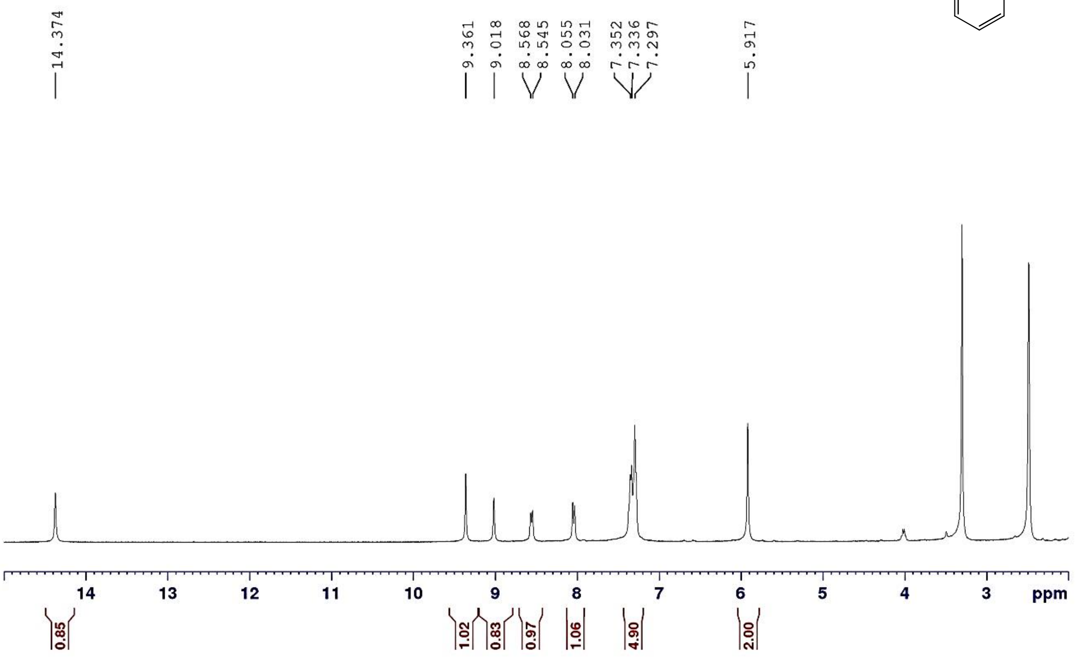

13C NMR compound 6a
DMSO

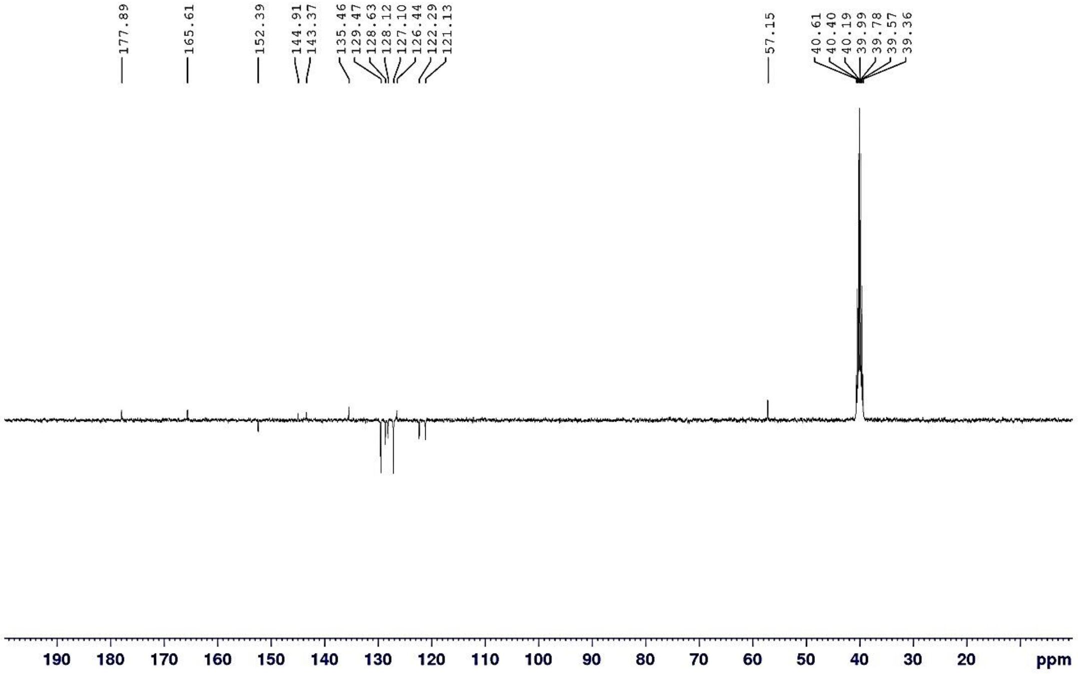

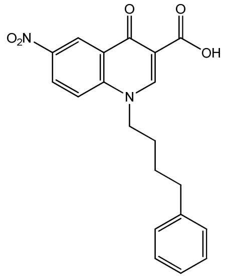

1H NMR compound 6d
DMSO

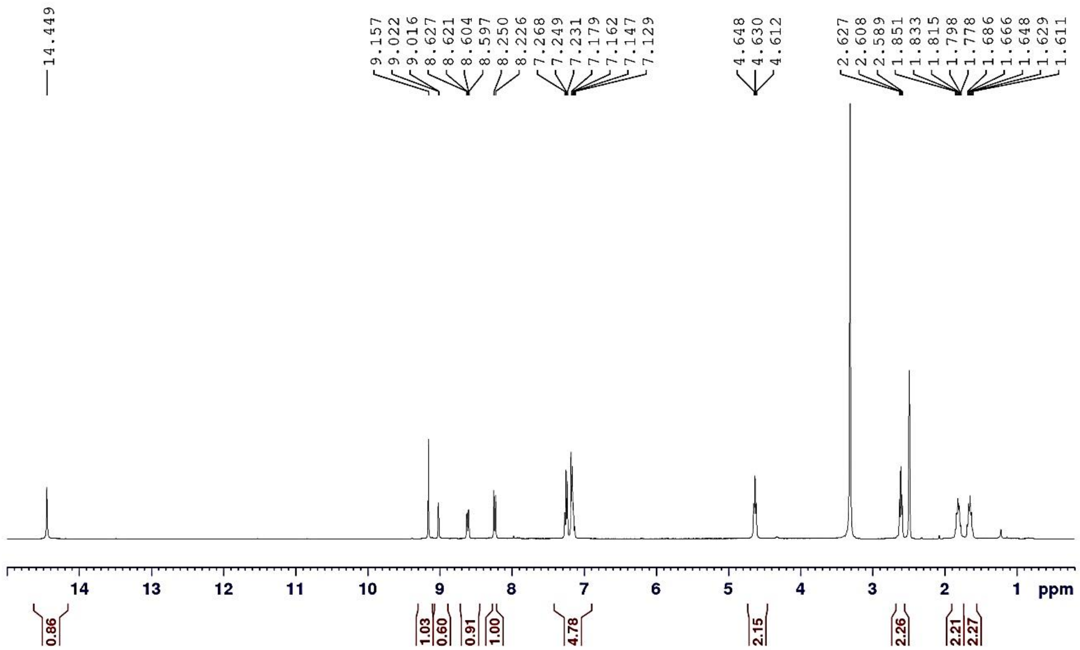

13C NMR compound 6d
DMSO

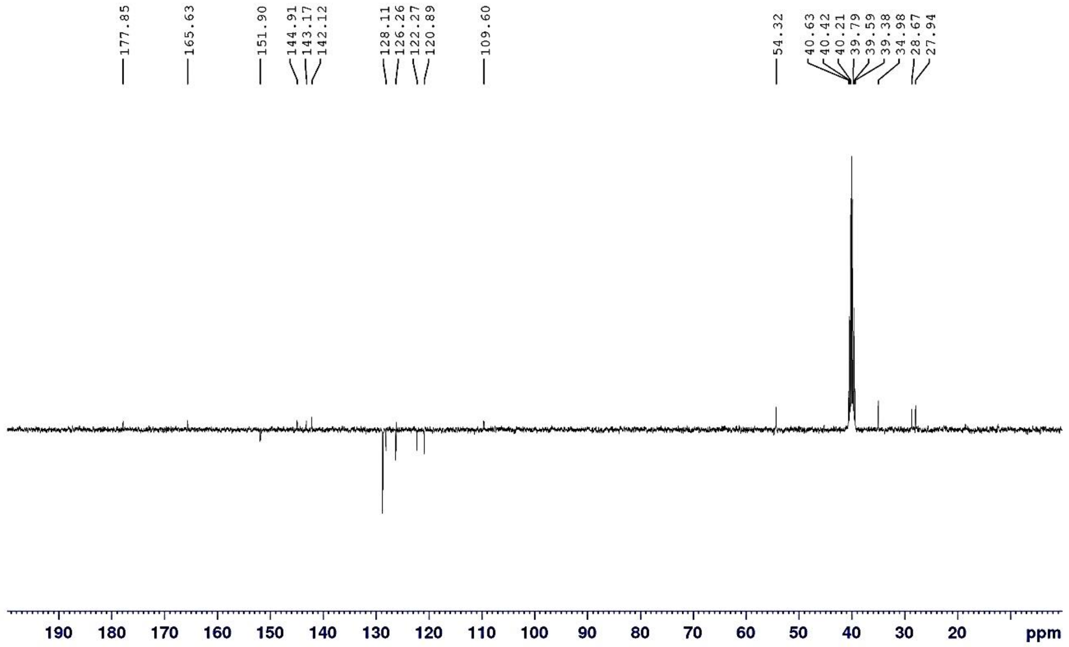

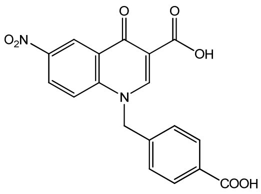

1H NMR compound 6g
DMSO

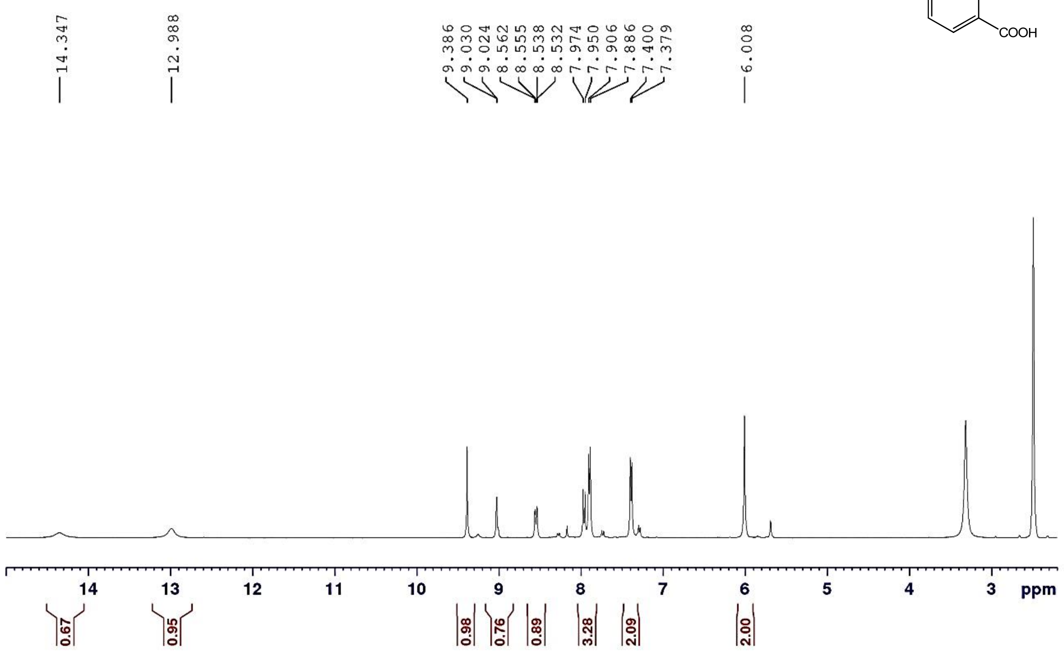

13C NMR compound 6g
DMSO

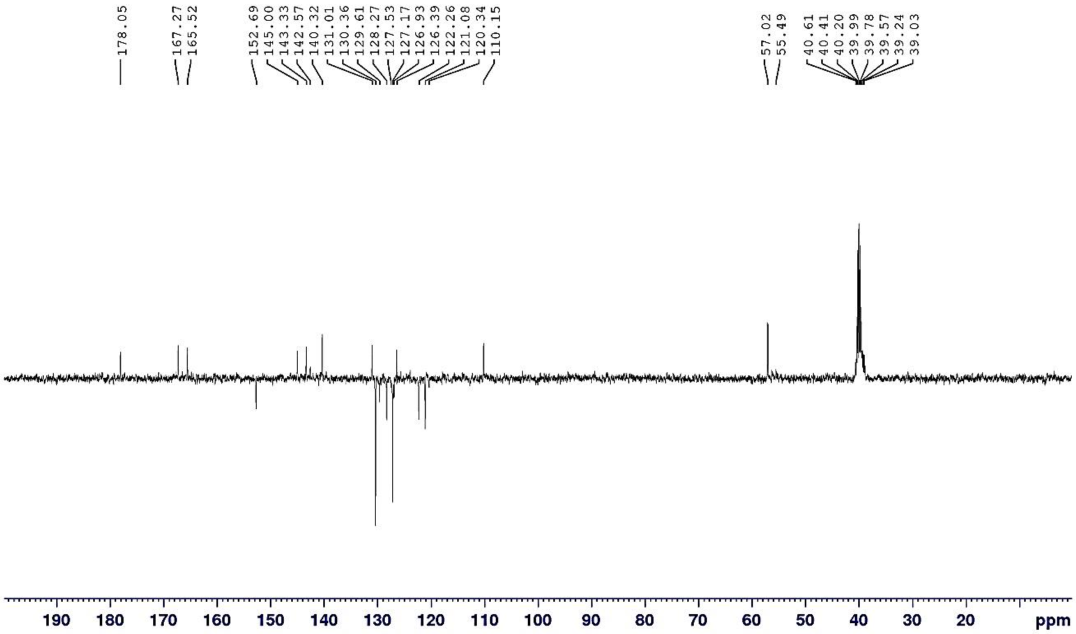

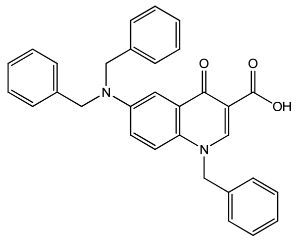

1H NMR compound 10
DMSO

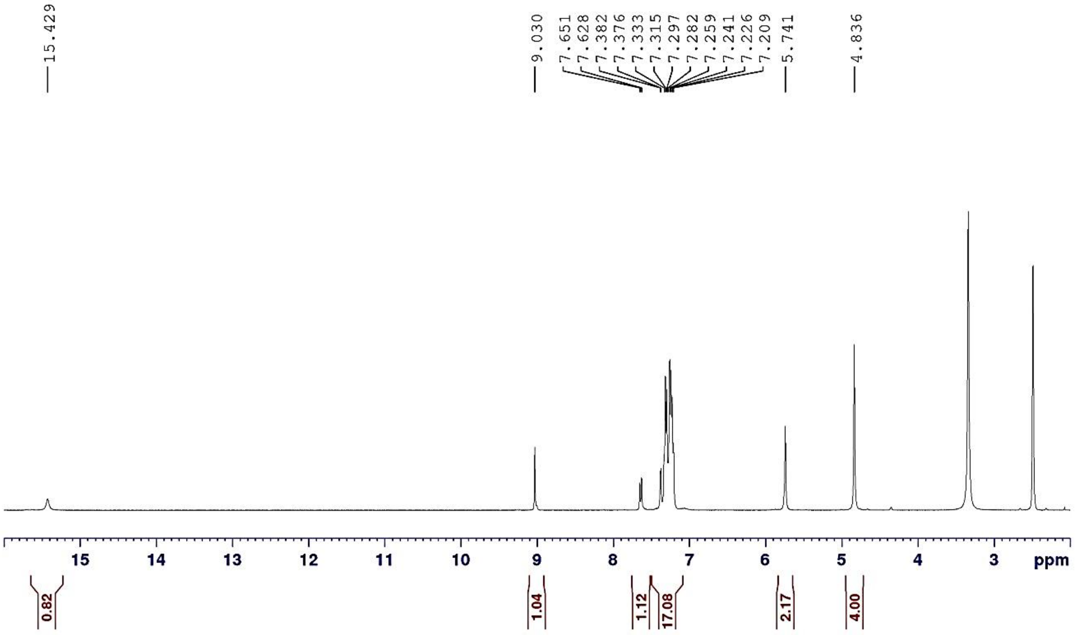

13C NMR compound 10
DMSO

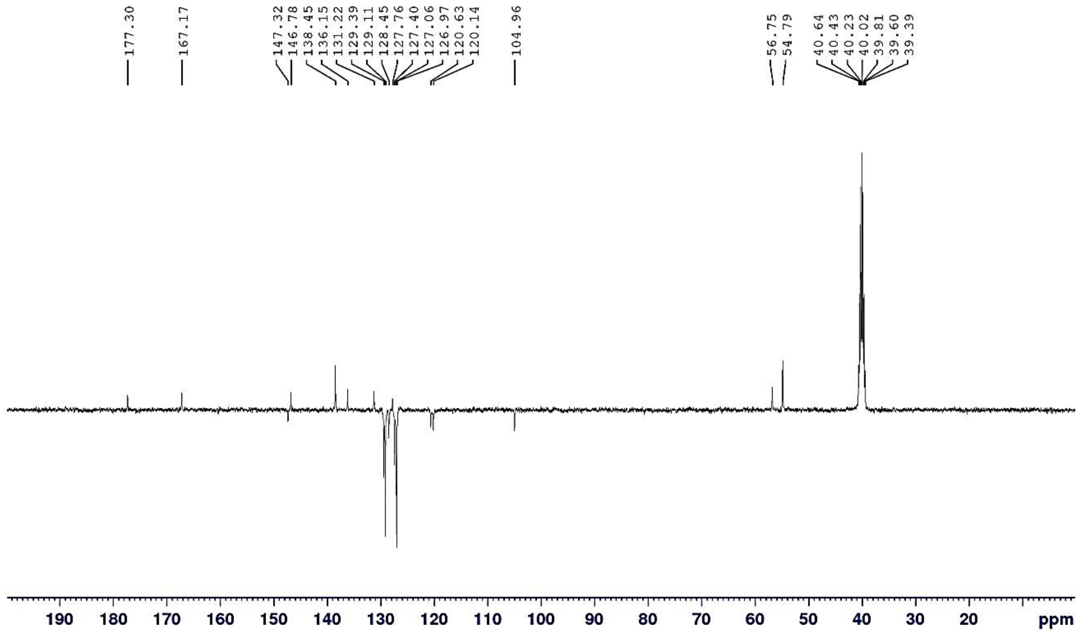

1H NMR compound **13c**
DMSO

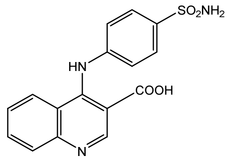

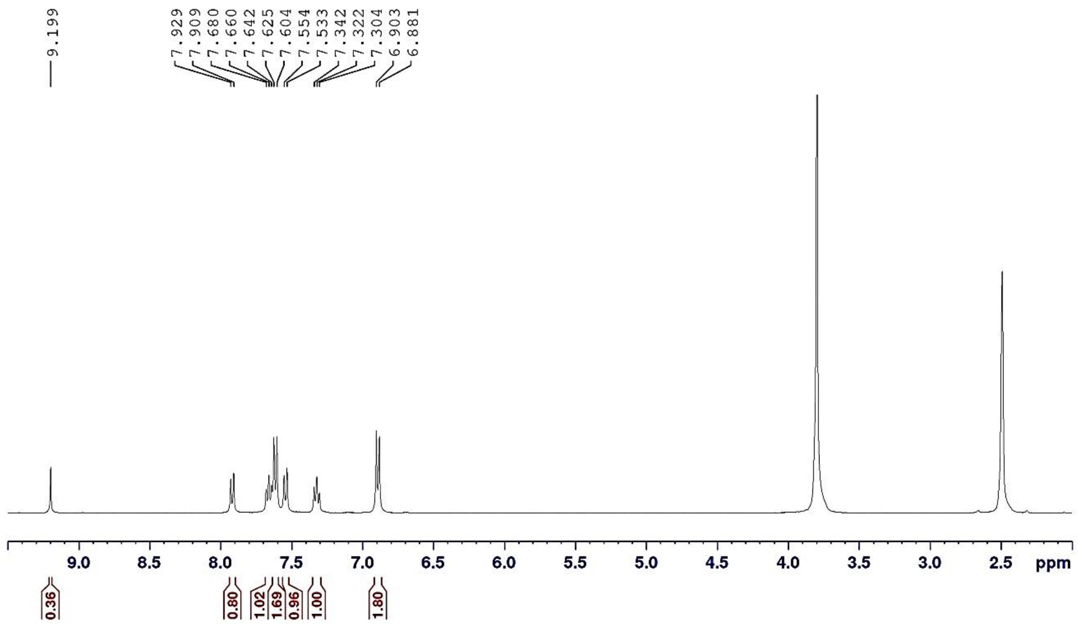

13C NMR compound **13c**
DMSO

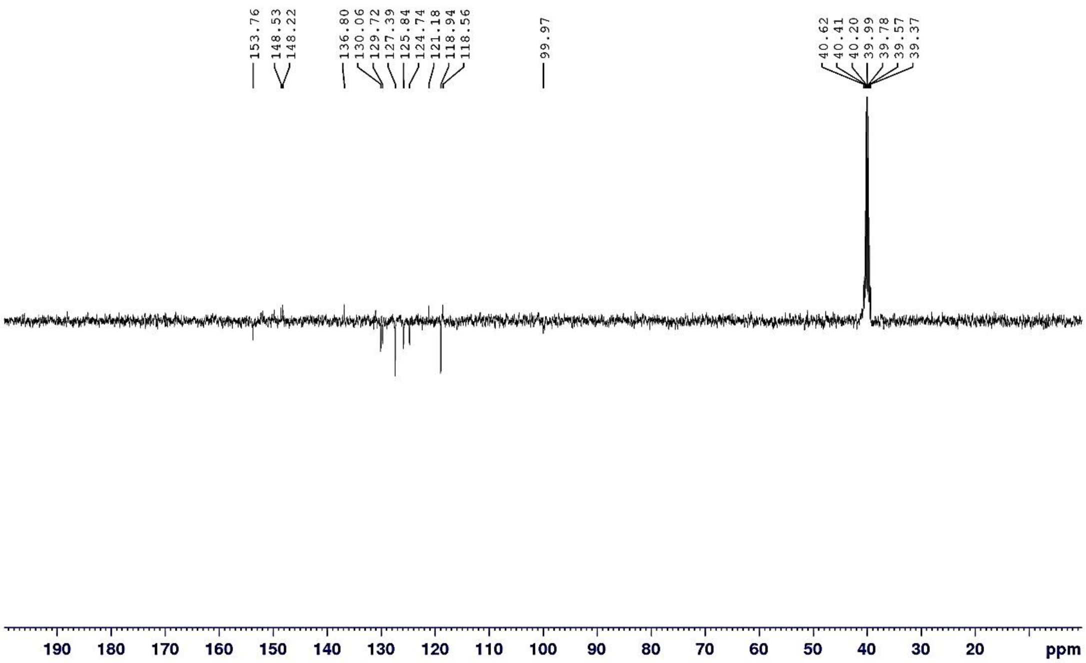

1H NMR compound **13d**
DMSO

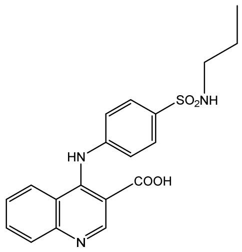

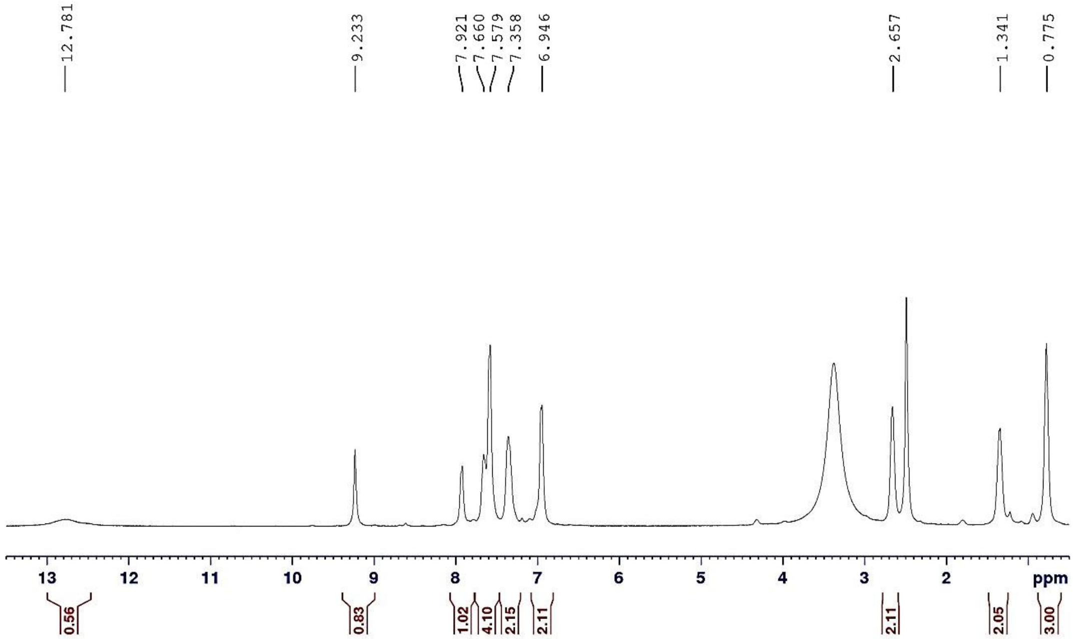

1H NMR compound **13d**
DMSO+D2O

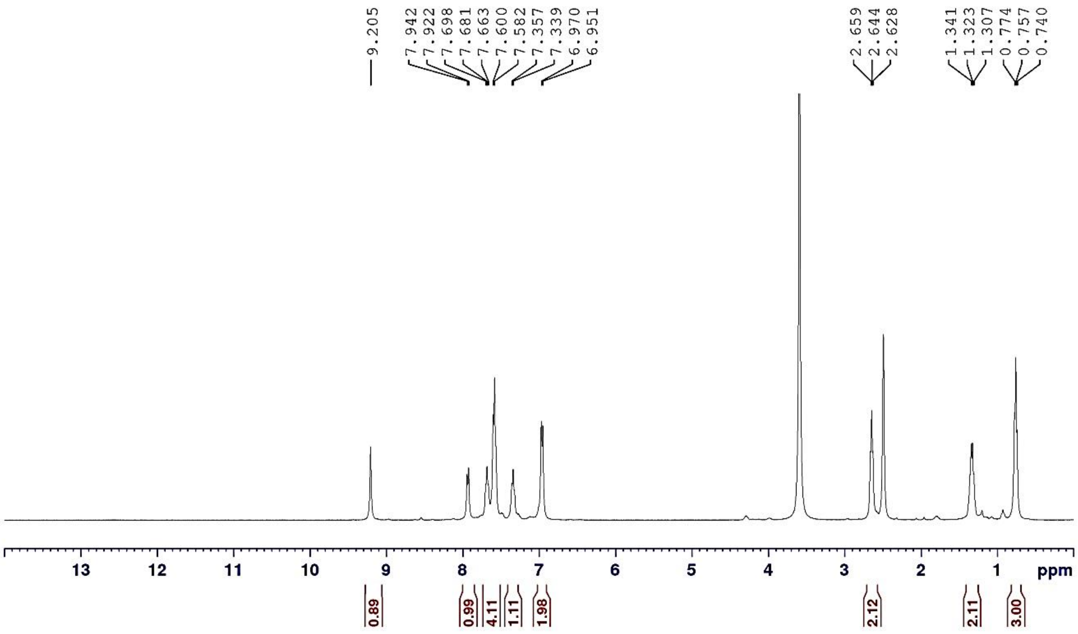

13C NMR compound **13d**
DMSO

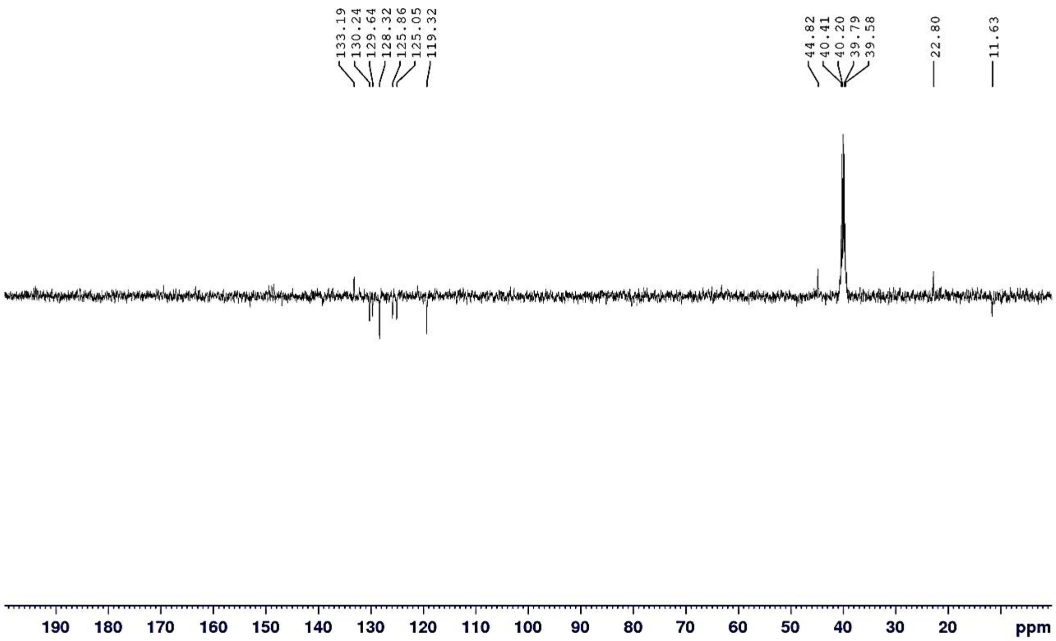

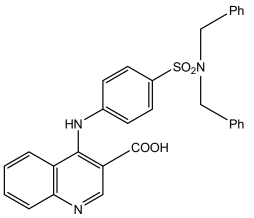

1H NMR compound **13f**
DMSO

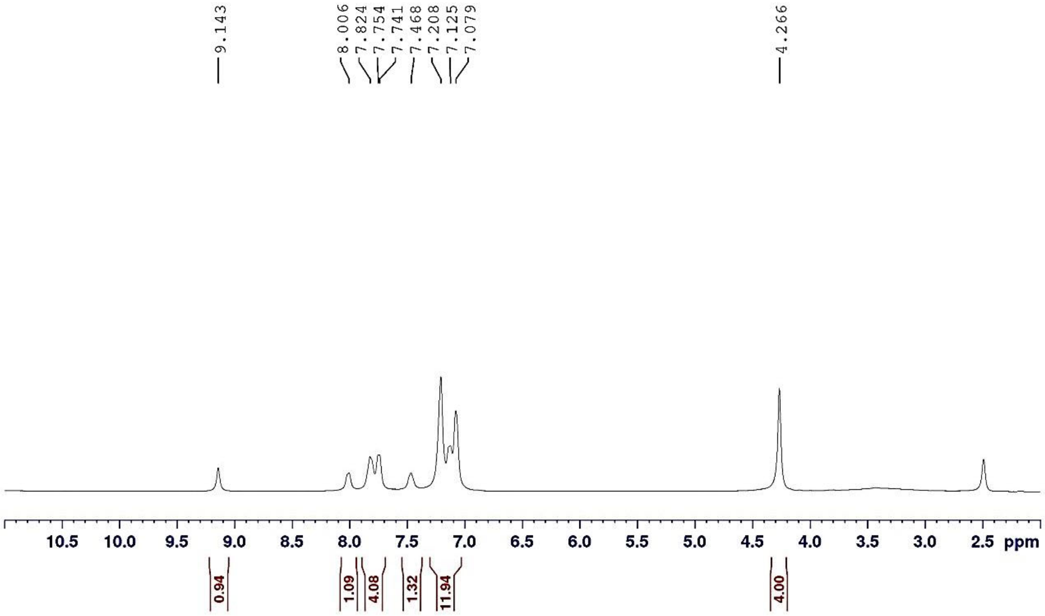

13C NMR compound **13f**
DMSO

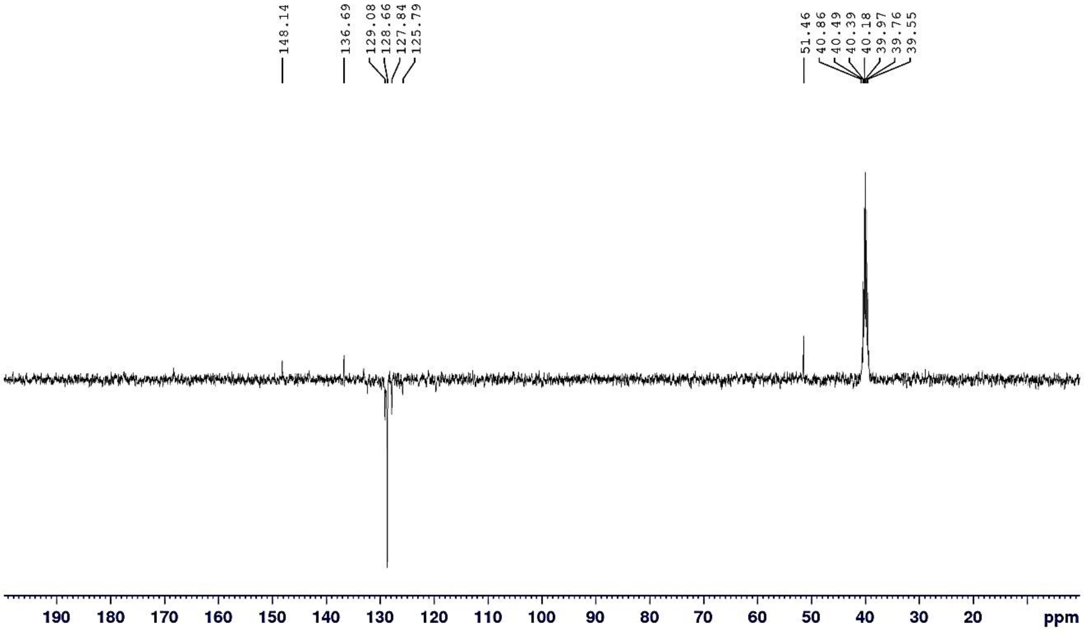

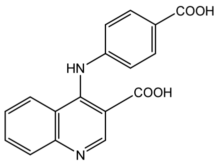

1H NMR compound **13g**
DMSO

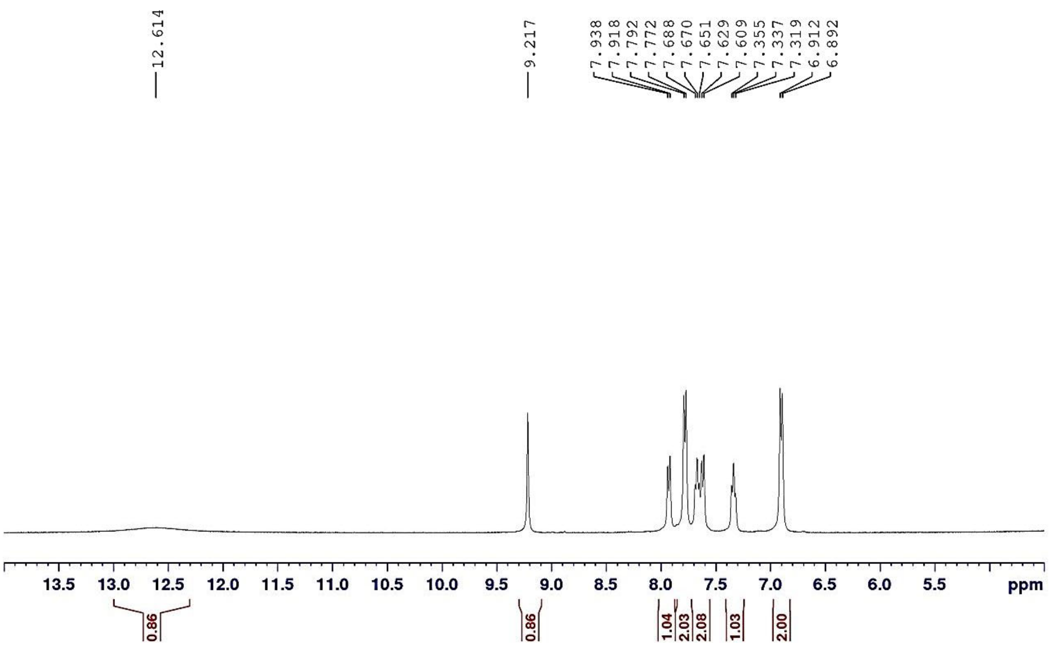

13C NMR compound **13g**
DMSO

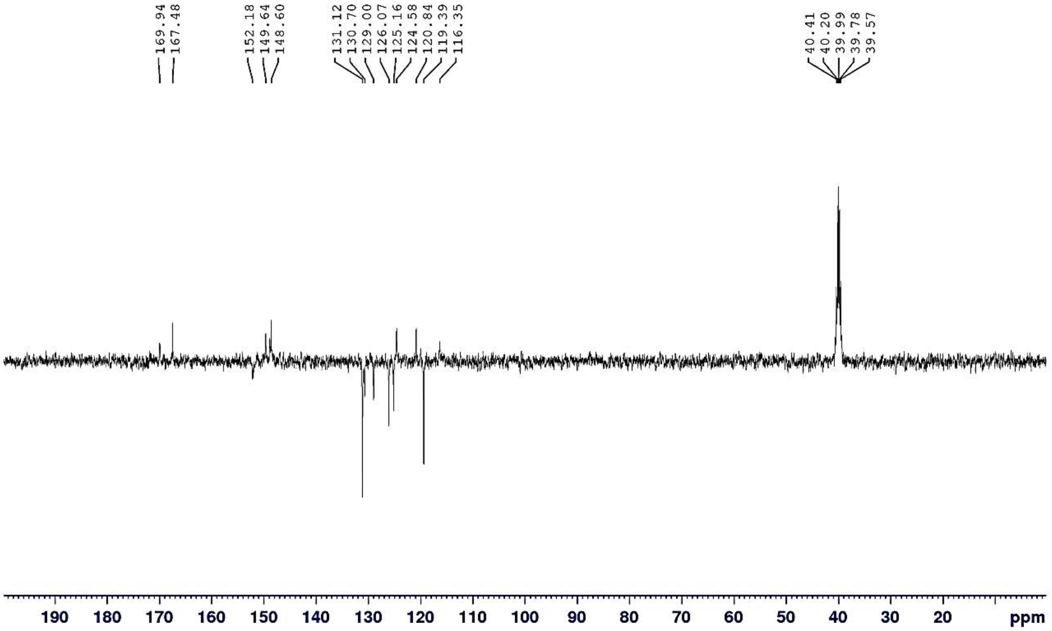

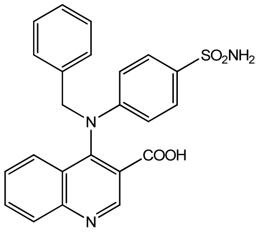

1H NMR compound 15
DMSO

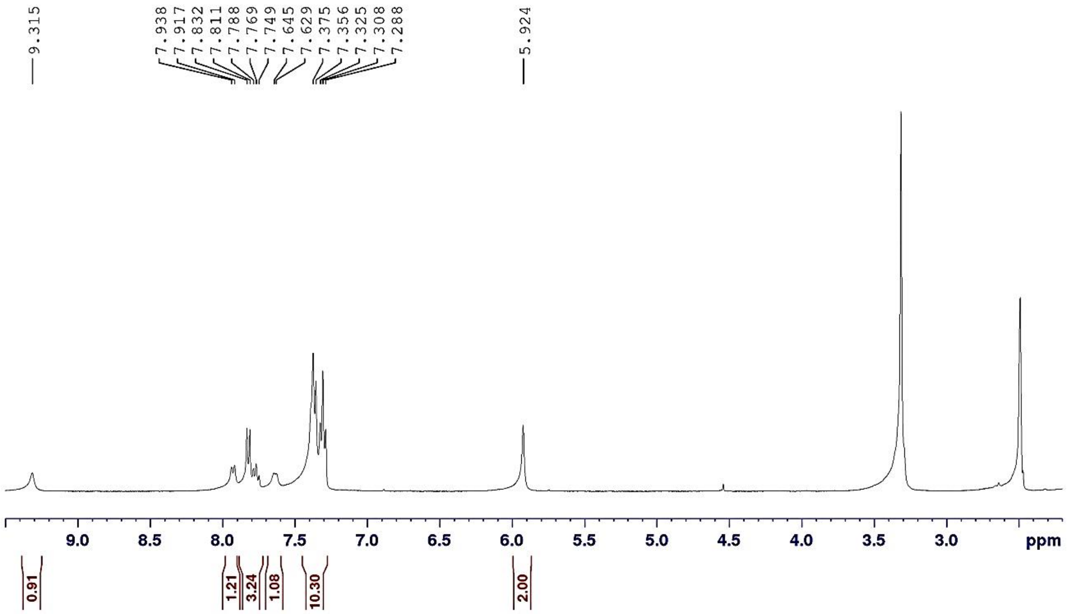

1H NMR compound 15
DMSO+D2O

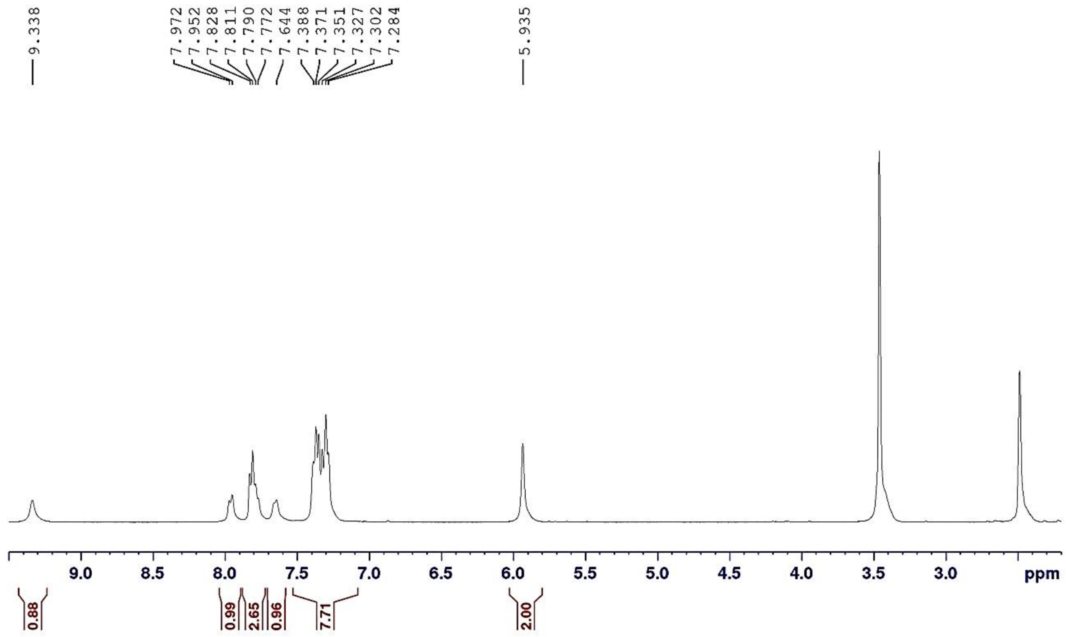

13C NMR compound 15
DMSO

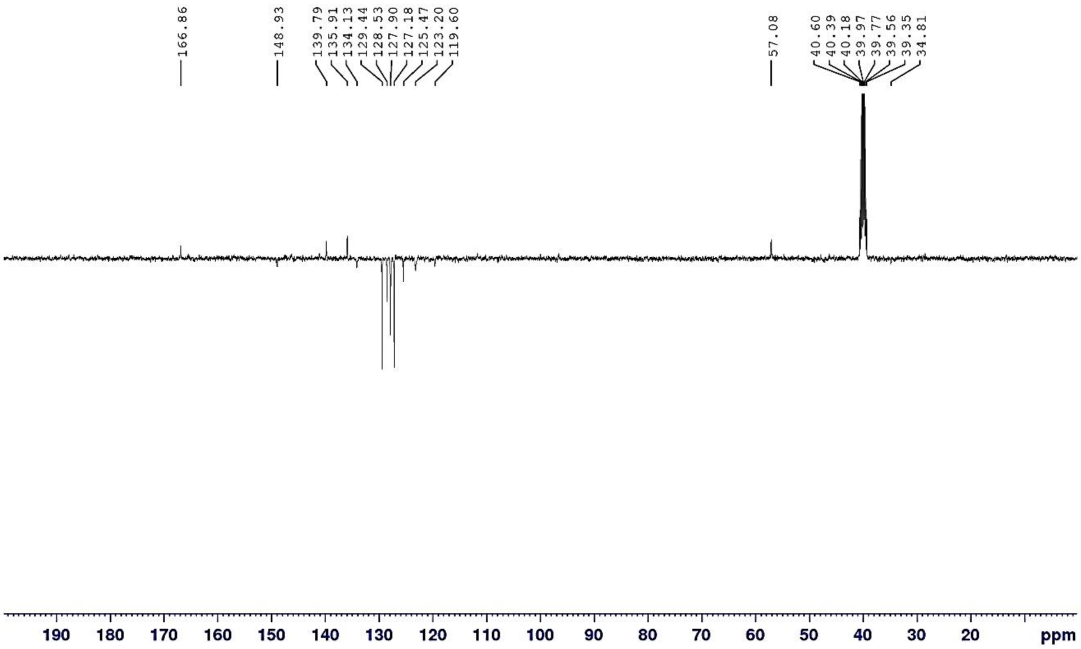

3. Chemical stability tests

Table S1: Stability of compounds and KEE in PBS and plasma

Comp.	PBS (min)	H-pl (min)
**4a**	>120	>120
**4b**	>120	>120
**4d**	>120	>120
**4e**	>120	>120
**4f**	>120	>120
**KEE Control**	-	119

Table S2: Elution gradient of mobile phase used for LC-MS/MS analyses

Time (min)	A (%)	B (%)	Flow (mL min^{-1})
0.00	60	40	0.25
2.50	10	90	0.25
4.50	10	90	0.25
4.51	90	10	0.25
6.50	90	10	0.25

solvent A: 5 mM of ammonium formate and 10mM of formic 875 acid in mQ water: acetonitrile 90:10 (v/v) solution;
solvent B: 5 mM of ammonium for-876 mate and 10mM of formic acid in mQ water: acetonitrile 10:90 (v/v) solution.

Table S3: MRM parameters

Compounds	Precursor ion (m/z)	Quantitation ion (m/z) [CE (V)]	Qualification ion (m/z) [CE (V)]
**IS**	455	165 [30]	-
**4a**	353	91 [15]	307 [15]
**4b**	367	105 [15]	321 [15]
**4d**	395	91 [15]	349 [20]
**4e**	409	217 [30]	363 [20]
**4f**	432	170 [25]	386 [15]

**KEE**	283	209 [15]	105 [30]

Table S4: Linear regressions data, linearity coefficients, LOD and LOQ values for each analyte

Compounds	Slope (PAR/ng ml^{-1})	Intercept (PAR)	R^2	y-SD LOD (ng mL^{-1})	y-SD LOQ (ng mL^{-1})
**4a**	0.102	0.151	0.998	2.5	7.5
**4b**	0.112	0.142	0.997	3.4	10.1
**4d**	0.115	0.191	0.997	3.4	10.2
**4e**	0.112	0.260	0.996	4.3	12.8
**4f**	0.029	0.031	0.999	2.0	6.0

Solution stability profiles

The solution stability profiles in PBS and human plasma were obtained by monitoring the variation of analyte concentration at different incubation times (0, 30, 60 e 120 min. at 37°C). They are reported in **Figures S1-S5**.

4a PBS/Hpl

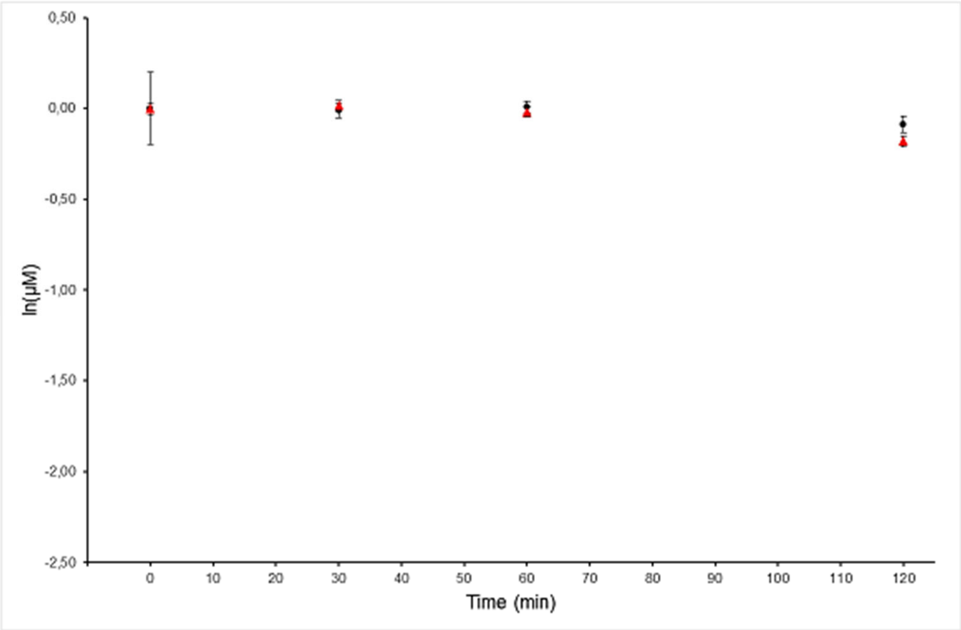

Figure S1. Degradation plots of compound **4a** in PBS (black spot) and human plasma (red triangle).

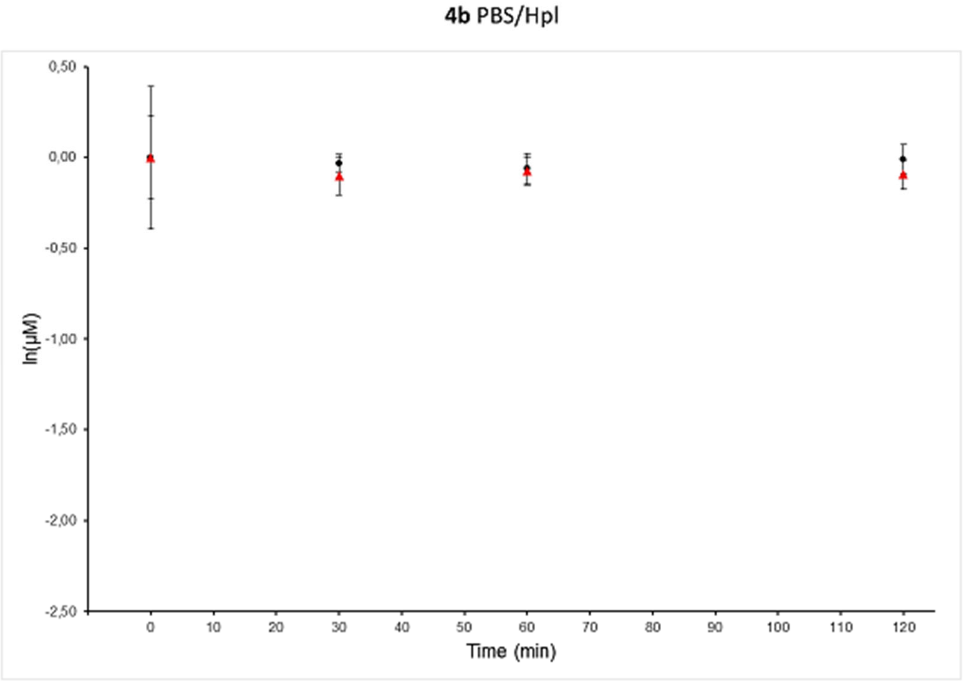

Figure S2. Degradation plots of compound **4b** in PBS (black spot) and human plasma (red triangle).

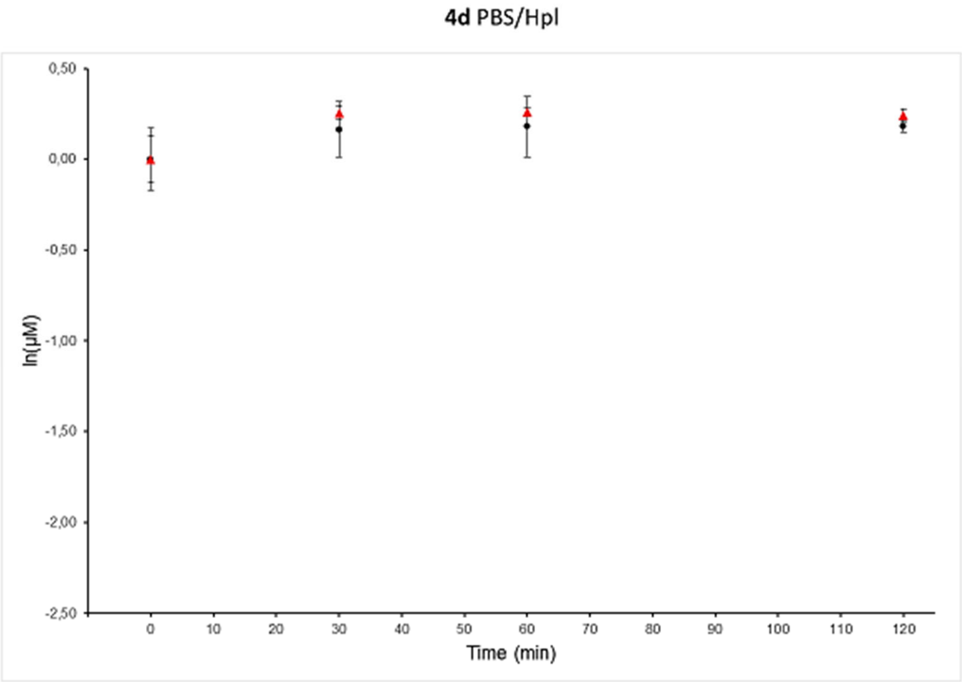

Figure S3. Degradation plots of compound **4d** in PBS (black spot) and human plasma (red triangle).

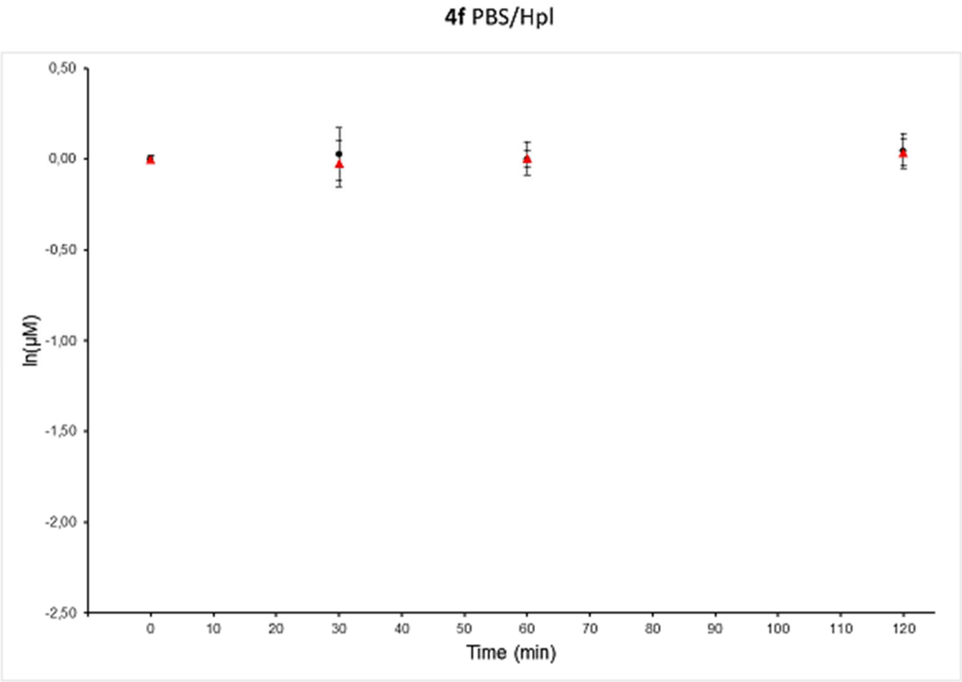

Figure S4. Degradation plots of compound **4f** in PBS (black spot) and human plasma (red triangle).

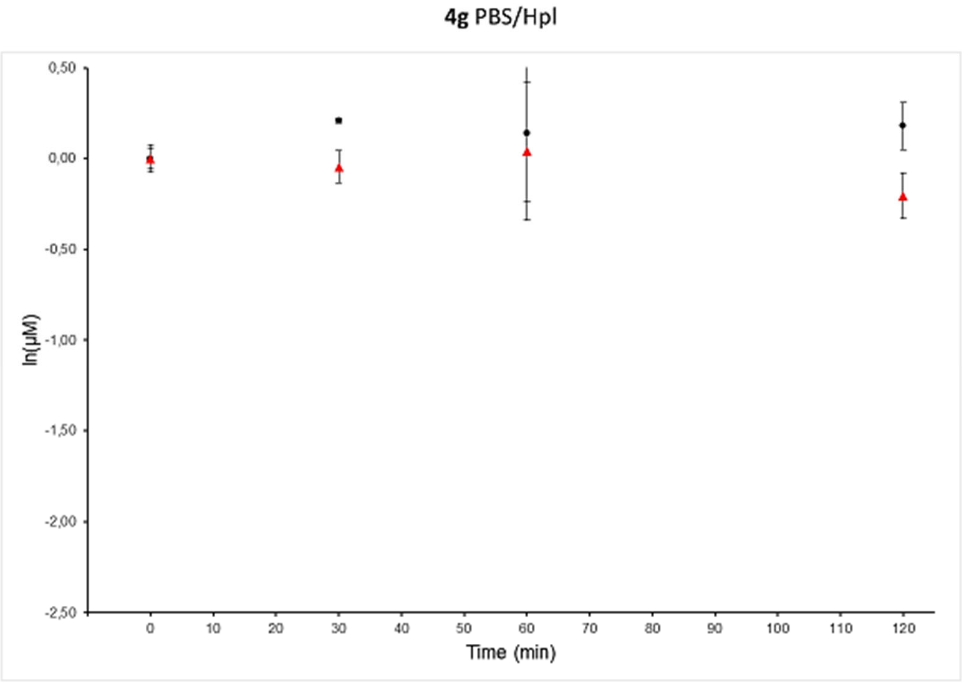

Figure S5. Degradation plots of compound **4g** in PBS (black spot) and human plasma (red triangle).

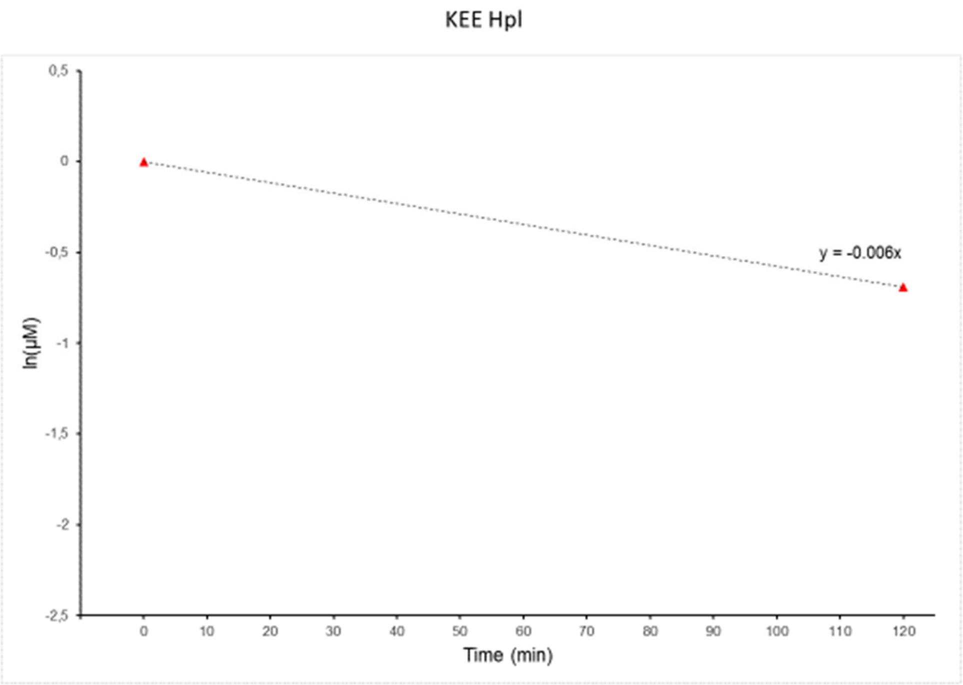

Figure S6. Degradation plot of Ketoprofen Ethyl Ester (KEE), reference compound, in human plasma.

4. **Single-Crystal X-ray Diffraction**

Table S5. Crystallographic data and refinement parameters for **4d**.

	**4d**
**empirical formula**	$C_{22}H_{22}N_2O_5$
**formula weight**	394.42
**T (K)**	100
**Crystal system, space group**	Triclinic, P-1
**λ (Å)**	1.54178
**Unit cell dimensions (Å, °)**	a = 10.451(2), α = 81.071(8)<br>b = 13.852(2), β = 71.945(8)<br>c = 15.030(2), γ = 69.602(9)
***V* (Å³)**	1936.2(5)
**Z, dcalc(g/cm³)**	4, 1.353
**μ (mm⁻¹)**	0.797
**F(000)**	832
**Reflections collected/unique**	26025 / 6301
**Data/paramenters**	6301 / 523
**Final R indices [I>2σ(I)]**	R1 = 0.0946, wR2 = 0.2538
**R indices all data**	R1 = 0.1355, wR2 = 0.3205
**GoF**	1.168

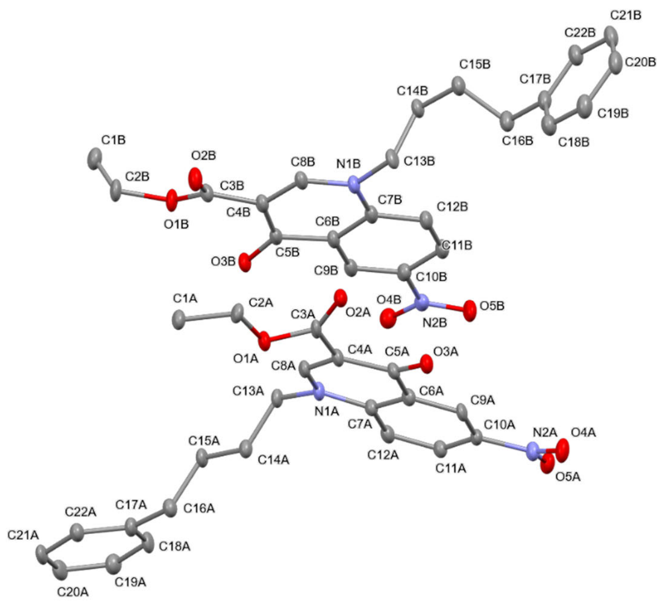

Figure S7. Ortep-3 view of the asymmetric unit of **4d**. Hydrogen atoms have been omitted for sake of clarity.

5. ADME assessment (Tables S6-S11)

Table S6. SwissADME calculated physiochemical properties.

Mol	Molecular Weight	LogP	TPSA
**6f**	403.372	1.3036	173.66 Å^2
**6g**	368.301	2.3544	142.42 Å^2
**13e**	427.526	4.4873	107.98 Å^2
**13g**	308.293	3.3748	99.52 Å^2

Table S7. SwissADME calculated lipophilicities parameters.

Mol	iLOGP	XLOGP3	WLOGP	MLOGP	Silicos-IT Log P	Consensus Log P
**6f**	0.42	1.87	2.91	-0.38	-0.77	0.81
**6g**	0.86	2.83	2.88	0.69	0.54	1.56
**13e**	3.14	4.57	5.57	0.91	2.67	3.41
**13g**	1.39	3.34	3.37	0.64	2.02	2.15

Table S8. SwissADME calculated water solubility parameters.

Mol	ESOL Log S	ESOL Solubility (mg/ml)	ESOL Solubility (mol/l)	ESOL Class	Ali Log S	Ali Solubility (mg/ml)	Ali Solubility (mol/l)	Ali Class
**6f**	-3.61	9.86E-02	2.44E-05	Soluble	-5.14	2.93E-03	7.27E-06	Moderately soluble
**6g**	-4.01	3.56E-02	9.66E-05	Soluble	-5.48	1.22E-03	3.32E-06	Moderately soluble
**13e**	-5.28	2.22E-03	5.20E-06	Moderately soluble	-6.75	7.64E-05	1.79E-07	Poorly soluble
**13g**	-4.11	2.41E-02	7.83E-05	Moderately soluble	-5.11	2.41E-03	7.82E-06	Moderately soluble

Table S9. SwissADME calculated pharmacokinetics parameters.

Mol	GI absorp.	BBB perm.	Pgp sub.	CYP2D6 substrate[a]	CYP3A4 substrate[a]	CYP1A2 inhibitor	CYP2C19 inhibitor	CYP2C9 inhibitor	CYP2D6 inhibitor	CYP3A4 inhibitor	log Kp (cm/s)
**6f**	Low	No	No	No	No	No	No	Yes	No	No	-7.43
**6g**	Low	No	No	No	No	No	No	Yes	No	No	-6.54

**13e**	Low	No	No	No	No	No	Yes	Yes	No	Yes	-5.54
**13g**	High	No	No	No	No	Yes	No	No	No	No	-5.81

[a] pkCSM (http://biosig.unimelb.edu.au).

Table S10. Swiss ADME calculated drug likeness parameters.

**Mol**	**Lipinski #violations**	**Ghose #violations**	**Veber #violations**	**Egan #violations**	**Muegge #violations**
**6f**	0	0	1, TPSA>140	1, TPSA>131.6	1, TPSA>150
**6g**	0	0	1, TPSA>140	1, TPSA>131.6	0
**13e**	0	0	0	0	0
**13g**	0	0	0	0	0

Table S11. pkCSM calculated excretion properties.

**Mol**	**Total Clearance Log(ml/min/kg)**	**Renal OCT2 substrate**
**6f**	0.89	No
**6g**	0.73	No
**13e**	0.70	No
**13g**	0.34	No

6. Table S12. Elemental analysis

Comp.	Formula (MW)	Anal. Calcd.				Anal. Found		
		C	H	N		C	H	N
**4b**	$C_{20}H_{18}N_2O_5$ (366.37)	65.57	4.95	7.65		65.83	4.97	7.68
**4c**	$C_{21}H_{20}N_2O_5$ (380.39)	66.31	5.30	7.36		66.57	5.32	7.39
**4d**	$C_{22}H_{22}N_2O_5$ (394.42)	66.99	5.62	7.10		66.72	5.59	7.07
**4e**	$C_{23}H_{24}N_2O_5$ (408.45)	67.63	5.92	6.86		67.36	5.90	6.83
**4f**	$C_{19}H_{17}N_3O_7S$ (431.42)	52.90	3.97	9.74		53.11	3.98	9.78
**4g**	$C_{20}H_{16}N_2O_7$ (396.35)	60.61	4.07	7.07		60.85	4.09	7.10
**5c**	$C_{21}H_{20}N_2O_5$ (380.39)	66.31	5.30	7.36		66.57	5.32	7.39
**5e**	$C_{23}H_{24}N_2O_5$ (408.45)	67.63	5.92	6.86		67.36	5.90	6.83
**6a**	$C_{17}H_{12}N_2O_5$ (324.29)	62.96	3.73	8.64		62.71	3.71	8.60
**6b**	$C_{18}H_{14}N_2O_5$ (338.31)	63.90	4.17	8.28		63.64	4.15	8.25
**6c**	$C_{19}H_{16}N_2O_5$ (352.34)	64.77	4.58	7.95		64.51	4.56	7.92
**6d**	$C_{20}H_{18}N_2O_5$ (366.37)	65.57	4.95	7.65		65.83	4.97	7.68
**6e**	$C_{21}H_{20}N_2O_5$ (380.39)	66.31	5.30	7.36		66.57	5.32	7.39
**6f**	$C_{17}H_{13}N_3O_7S$ (403.37)	50.62	3.25	10.42		50.42	3.24	10.38
**6g**	$C_{18}H_{12}N_2O_7$ (368.30)	58.70	3.28	7.61		58.46	3.27	7.58
**8**	$C_{17}H_{14}N_2O_3$ (294.30)	69.38	4.79	9.52		69.66	4.81	9.56
**9**	$C_{33}H_{30}N_2O_3$ (502.60)	78.86	6.02	5.57		78.54	5.99	5.54
**10**	$C_{31}H_{26}N_2O_3$ (474.55)	78.46	5.52	5.90		78.77	5.54	5.92
**12a**	$C_{15}H_{13}N_3O_2S$ (299.35)	60.18	4.38	14.04		60.42	4.40	14.09
**12b**	$C_{16}H_{12}N_2O_2$ (264.28)	72.72	4.58	10.60		72.43	4.56	10.55
**12c**	$C_{18}H_{17}N_3O_4S$ (371.41)	58.21	4.61	11.31		58.44	4.63	11.35
**12d**	$C_{21}H_{23}N_3O_4S$ (413.49)	61.00	5.61	10.16		61.24	5.63	10.20
**12e**	$C_{24}H_{29}N_3O_4S$ (455.57)	63.28	6.42	9.22		63.53	6.44	9.25
**12f**	$C_{32}H_{29}N_3O_4S$ (551.66)	69.67	5.30	7.62		69.39	5.38	7.59
**12g**	$C_{19}H_{16}N_2O_4$ (366.35)	67.85	4.80	8.33		67.58	4.82	8.36
**13c**	$C_{16}H_{13}N_3O_4S$ (343.36)	55.97	3.82	12.24		55.75	3.80	12.19
**13d**	$C_{19}H_{19}N_3O_4S$ (385.44)	59.21	4.97	10.90		59.45	4.99	10.94
**13e**	$C_{22}H_{25}N_3O_4S$ (427.52)	61.81	5.89	9.83		61.56	5.87	9.79
**13f**	$C_{30}H_{25}N_3O_4S$ (523.61)	68.82	4.81	8.03		68.54	4.79	8.00
**13g**	$C_{17}H_{12}N_2O_4$ (308.29)	66.23	3.92	9.09		66.49	3.93	9.12
**14**	$C_{25}H_{23}N_3O_4S$ (461.53)	65.06	5.02	9.10		65.32	5.04	9.13

**15**	$C_{23}H_{19}N_3O_4S$ (433.48)	63.73	4.42	9.69		63.47	4.40	9.65
